# A Public Health Opportunity Found in Food Waste

**DOI:** 10.5888/pcd14.160596

**Published:** 2017-11-02

**Authors:** Shreela V. Sharma, Mudita Upadhyaya, Gregory Bounds, Christine Markham

**Affiliations:** 1 The University of Texas School of Public Health, Michael & Susan Dell Center for Healthy Living, Houston, Texas; 2 The University of Texas School of Public Health, Department of Health Promotion and Behavioral Sciences, Houston, Texas

## Abstract

A food waste problem coexists with food insecurity and obesity. Brighter Bites, a school-based food cooperative program, successfully channels primarily donated produce to low-income communities and provides nutrition education, creating an increased demand for and intake of fruits and vegetables. We present the framework used in Brighter Bites and results of operationalizing this framework during 3 years of implementation in Houston, Texas. Results demonstrated that, during 2013 through 2016, more than 12,500 families enrolled in Brighter Bites for 16 weeks in the school year. More than 90% of the produce was donated. Each week, families received on average 54 to 61 servings of fresh produce with the average cost of produce being $2.53 per family per week. Of those parents who completed the process surveys, more than 80% reported the produce to be effective in improving their children’s diet. Brighter Bites demonstrates a successful model to address food waste and improve dietary habits of underserved families.

## Introduction

In the United States, hunger, food waste, and chronic disease are intricately linked. The United States has an epidemic of food insecurity and obesity that coexists in the same population (ie, low-income families on a budget) (1,2). Moreover, fruits and vegetables, which are linked to improving health and preventing chronic disease (3,4), are also perishable and commonly wasted. Food waste in the United States occurs primarily at 3 levels — production (eg, farm), retail (eg, grocery store), and consumer or household (5,6). The US Department of Agriculture (USDA) reports more than 40 billion pounds of postharvest loss of produce, estimated at more than $50 billion each year (5,6). The US per capita food waste has increased by approximately 50% since 1974 (7). 

Concurrently, despite the progressive increase in the US per capita food supply during the past 4 decades, the United States has more than 42.2 million food-insecure Americans who lack regular access to food, especially healthy foods such as fruits and vegetables (8). Moreover, approximately 60% of children eat fewer fruits and 93% eat fewer vegetables than recommended (9). Food insecurity, especially among children, could be invisible in the United States as a result of the simultaneous high intake of unhealthy foods and beverages that lead to obesity. These issues present a contradictory state in that a major food waste problem coexists with food insecurity and obesity. 

Although food waste has been quantified extensively in the literature (5,6), publications related to efforts that decrease the waste of healthy foods while promoting efforts to mitigate food insecurity and chronic disease among communities struggling with these issues are minimal. We aim to add to the literature by proposing a framework to mitigate food waste and convert it into a public health opportunity by using the Brighter Bites program as an example to operationalize this framework. We also present data from 3 years of implementation of the Brighter Bites program in Houston, Texas, to illustrate the feasibility and acceptability of operationalizing this framework.

## Converting Food Waste Into a Public Health Opportunity — The Brighter Bites Case Study

In Texas, children’s consumption rates of fruits and vegetables are among the lowest in the country (10), while obesity and food insecurity rates among adults and children are among the highest (11). In response to these epidemics, in 2012, a program called Brighter Bites was launched in Houston, Texas. Brighter Bites aims to tackle food waste and improve access to fresh produce and nutrition education among underserved children and their families. The Figure demonstrates the proposed framework of Brighter Bites as a solution to address food waste and convert it into a health promotion opportunity.

**Figure:**
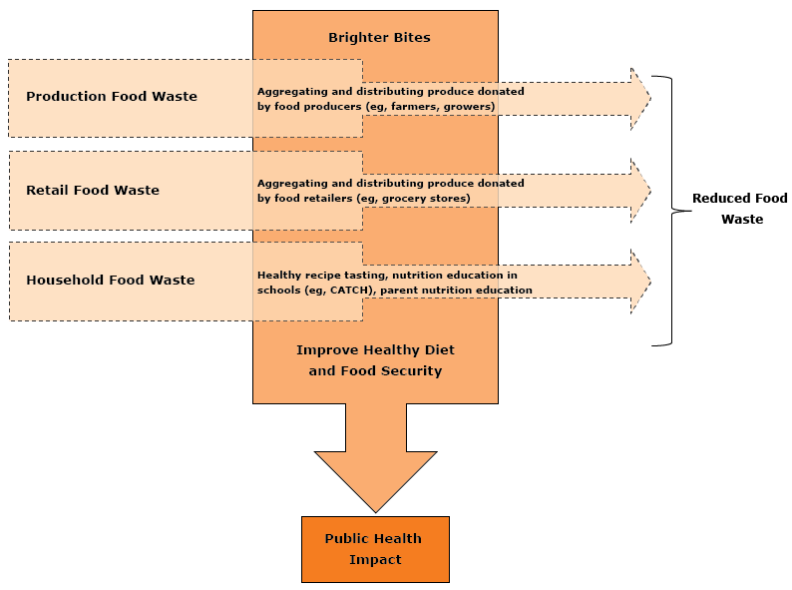
Brighter Bites framework for addressing food waste and improving dietary habits, Houston, Texas, 2013–2016. Abbreviation: CATCH, Coordinated Approach to Child Health.

## Brighter Bites Intervention

Brighter Bites is a theoretically grounded school-based, preschool-based, and after school–based program that uses constructs from social cognitive theory to create a demand for and improve the intake of fruits and vegetables among low-income children and their families (12,13). The formula consists of 1) produce distribution (50–60 servings per family per week) using primarily donated produce, 2) nutrition education in school and for parents, and 3) a healthy recipe tasting, implemented using a food cooperative (co-op) concept for 16 weeks during the school year and 8 weeks during the summer (13). As part of the Brighter Bites food co-op, the program engages parent volunteers in assisting with program implementation at their site, including the weekly bagging and distribution of the produce. Primarily donated produce is procured, stored, and distributed using the existing infrastructure of local food banks. Eligibility for Brighter Bites is based on site-level socioeconomic status (eg, >75% of children on free or reduced-price lunch programs in schools, Head Start programs). When parents pick up their children from school, each family receives 2 bags of produce for the week, family members taste a recipe made by Brighter Bites staff using a produce item in the bag, and families receive the recipes and tips on how to prepare it at home. Parents also receive 2 nutrition handbooks that provide information on food preparation and storage techniques, menu planning, healthy recipes, healthy grocery shopping, and label reading. All parent materials are provided in English and Spanish. Participating sites’ teaching and physical education staff are trained in implementing the Coordinated Approach to Child Health (CATCH), an evidence-based coordinated school health program proven to reduce obesity prevalence among low-income children (14). Moreover, the Brighter Bites website has additional nutrition resources including tip sheets and recipes for families to access. Brighter Bites is implemented at no cost to the site or families. The commitment is for parents or family members to engage in the weekly co-op to assist with bagging and distributing the produce. School, preschool, or community sites interested in participating in the program fill out a Brighter Bites membership application. After this step, at the start of the school year or summer, families from the participating site opt in to the program. Families from participating sites may re-enroll in to the program each school year. 

Brighter Bites started organically in 2012 with 150 families in a single school, and the program has distributed more than 10 million pounds of primarily donated produce to more than 20,000 low-income children and their families across more than 100 schools, Head Starts, YMCAs, and community centers in Houston, Dallas, and Austin, Texas (www.brighterbites.org). Brighter Bites achieves this aim by collaborating with for-profit food distribution companies that are aggregating produce directly from farmers across the country and local grocery companies that send produce to local food banks, which then transport it to the Brighter Bites sites. In partnership with The University of Texas Health Science Center at Houston School of Public Health, Brighter Bites collects process evaluation data to monitor program dosage, reach, fidelity, and acceptability from families enrolled in the program. Each week Brighter Bites coordinators and implementation staff complete surveys to provide data on produce distribution (what was distributed and how much), parent and other volunteer attendance, recipe tasting information (type of recipe), and education implementation at each site. Attendance rosters provide data on weekly produce pick-up by each family; parents complete surveys twice a year on acceptability, usage, and perceived effectiveness of Brighter Bites program components. The cost of providing produce per family per week is obtained from the food banks that aggregate and deliver the produce to the Brighter Bites sites. Qualitative data in the form of focus groups and other methods such as Photovoice with the parents further inform program development and evaluation (15,16). We used the USDA nutrient database to calculate the number of servings distributed to families from the quantities and types of produce provided (17).

## Impact of Brighter Bites

As part of this case study, we assessed the process evaluation data collected during 3 school years of implementation of Brighter Bites in Houston (Table). Results indicated that enrollment of sites and families in the Brighter Bites program consistently increased during the 3 school years in Houston. During the 2013–2014 school year, 9 sites had an average of 1,224 families enrolled; during the 2014–2015 school year, 20 sites had an average of 2,625 families enrolled; and during the 2015–2016 school year, 30 sites had an average of 8,216 families enrolled. Enrollment for the 2016–2017 school year in Houston is now 11,500. On average, families received 53.6 to 61.3 servings of 8 to 12 different types of fruits and vegetables each week during the 3 school years. Cost of the produce decreased with increasing number of families served, starting with $2.67 in the 2013–2014 school year to $2.29 in the 2015–2016 school year. Retail cost of the produce determined by using randomly sampled bags at local grocery stores in Houston was estimated to be $35 to $40. Across the 3 school years, more than 90% of the produce distributed was donated produce procured from production (eg, farmers) and retail (eg, grocery stores) sources locally and nationwide. On average, 4 to 10 parents volunteered each week in the Brighter Bites co-ops to assist with the bagging and distribution of the produce.

**Table:** Brighter Bites Process Evaluation Data for 3 School Years, Houston, Texas, 2013–2016

Characteristic	2013–2014	2014–2015	2015–2016
**Mean no. of sites and families enrolled in the program during fall and spring**	9 sites; 1,224 families	20 sites; 2,625 families	30 sites; 8,216 families
**Food redistribution**			
Mean no. of servings of fruits and vegetables provided per family per week	60.1	53.6	61.3
Average cost of produce per family per week, $^a^	2.67	2.63	2.29
Percentage of donated produce	NA	96.5	97.9
**Mean no. of families who reported that the education and diet impact facilitated healthy choices^b^**	438	687	2,057
**Families ate all or most of provided produce, %**			
Fruits	93.6	97.1	96.5
Vegetables	88.6	94.8	92.9
**Perceived effectiveness of the fruit provided to influence child’s intake of fruits and vegetables^c^**	90.7	94.3	84.6
**Perceived effectiveness of the vegetables provided to influence child’s intake of fruits and vegetables^c^**	86.4	92.7	83.3
**Perceived effectiveness of the nutrition booklet to influence child’s fruits and vegetable intake^c^**	77.8	85.1	74.7
**Perceived effectiveness of the recipe cards to influence child’s fruits and vegetable intake^c^**	79.0	85.4	74.2
**Self-reported savings per family, $**	31.05	31.48	29.80

Abbreviation: NA, not available.

a Average cost of produce per family per week is the total cost of produce procurement and delivery costs per week per site (as obtained from the food bank) divided by the total number of families enrolled in the program.

b Mean number of families who responded on the process evaluation survey at the end of 8 weeks and 16 weeks of the Brighter Bites program.

c Percentage of participants who reported activity as “highly effective.”

Results from the parent surveys completed at the end of 8 weeks and 16 weeks of programming demonstrate that, consistently across the 3 school years, more than 90% of the parents reported using all or most of the fruits and more than 85% reported using all or most of the vegetables provided as part of Brighter Bites. When asked about the perceived effectiveness of each of the program components, more than 83% of the parents reported the fruits and vegetables to be effective in improving their child’s intake. The perceived effectiveness was higher (>85%) in the 2013–2014 and 2014–2015 school years than during the 2015–2016 school year. Furthermore, most parents (>70%) also reported the nutrition handbooks and recipe cards to be effective; however, the perceived effectiveness of the education materials was lower than that of the produce provided. Moreover, across the 3 school years, parents reported saving $29.80 to approximately $30 per week on their grocery bill for the duration of the program.

## Addressing Food Waste and Creating Public Health Opportunity

The framework of channeling primarily donated produce into low-income communities and combining it with education on a consistent basis provides an opportunity to mitigate food waste and increase intake of healthful food among underserved families. Our case study of the Brighter Bites program demonstrates a successful operationalization of this framework. Brighter Bites is giving parents living on a limited income — who traditionally have low access to fresh produce or who have been afraid of buying fruits and vegetables because they either do not know how to prepare them or cannot manage the financial risk that their children won’t eat it — a “free trial” to practice cooking and eating healthy foods with their children. Brighter Bites also operationalizes the Centers for Disease Control and Prevention’s Whole School, Whole Community, Whole Child approach (18) by implementing the program using a coordinated school health model, engaging the parents and community using a food co-op concept, and providing consistent access to fresh produce and nutrition education in school and for parents through the school year. For a family of 4, the weekly 50 to 60 servings of produce provide an additional 2 servings per family member per day. By partnering with food banks to procure donated produce and channeling it purposefully with continuity into underserved communities, Brighter Bites is addressing the issue of food waste at the production and retail levels. By combining this produce distribution with ongoing in-school and parental nutrition education consisting of healthy cooking tips and tools, information about food storage and food safety, and healthy recipes, Brighter Bites is teaching families how to use the produce, thus reducing food waste at the household or consumer level, and creating opportunities for families to practice healthy behaviors. 

Results of previous studies demonstrate high fidelity and feasibility of implementation and acceptability of program components among families (13,15). A 2-year study among 760 first grade children and their parents during the 2013–2015 school years demonstrated that, compared with those not receiving Brighter Bites, children and their parents receiving Brighter Bites had a significant increase in intake of fruits and vegetables, and children reported consuming fewer calories from added sugars than did those who did not receive the program (13). Also, the study reported improvements in the home environment, including increase in frequency of cooking at home, increased usage of nutrition facts labels to make grocery purchasing decisions, and having more fruits and vegetables available at home during meals, compared with families who did not receive the program (13). Participating families on average attended 7 of the 8 Brighter Bites weekly produce distributions during the fall and spring semesters (13). Qualitative data from focus groups and other methods such as Photovoice support these findings that Brighter Bites enables parents to provide their children with opportunities to practice healthy eating and to introduce new flavors and try new recipes and foods at home, and the program gives them the financial flexibility that was previously lacking (15,16).

The framework of Brighter Bites to address food waste is achieved by leveraging existing infrastructures of nonprofit and for-profit partnerships to obtain the produce donations and distribute them into low-income communities. Brighter Bites is partnering with local food banks in Houston, Dallas, and Austin, Texas, that are aggregating and warehousing the produce and then distributing it to Brighter Bites locations. Through these critical partnerships, Brighter Bites is able to deliver the produce directly and consistently to underserved families while teaching them how to use it. Furthermore, the program is creating opportunities for children to practice these healthy behaviors while at school and, in doing so, successfully linking the school and the home — the 2 environments where children spend most of their time.

## Challenges to Operationalizing the Framework

These strengths notwithstanding, operationalizing the framework has several challenges. The first challenge is sourcing and procuring the produce. Brighter Bites, with the support of food banks, is able to obtain a small percentage of produce donations that would otherwise be discarded at the production and retail levels because of limitation of funding and resources available. For example, there is a significant cost at the food production level associated with picking, packing, and shipping of produce. Incentives to farmers are needed to offset these costs to pick the lower-grade produce and ship it to local food banks. Another challenge is quality of the produce procured. Approximately 10% of the produce that is procured at the food bank is at the end of its shelf life. This is addressed in 2 ways. Food bank and Brighter Bites staff conduct daily quality control checks of the produce at the food bank before it is distributed, and families are educated each week on which produce to eat first, depending on its shelf life. A related challenge is obtaining a variety of produce. The program sends home at least 8 to 10 different kinds of produce each week, which requires the staff to work closely with the food bank inventory managers on a daily basis to ensure the variety. Also, a challenge with participating schools is successful implementation of the CATCH coordinated school health program. Ongoing engagement and provision of technical support is needed to maintain CATCH implementation in the schools and ensure robustness of the nutrition education component. Finally, the parent survey data collected as part of process evaluation are self-reported and could be influenced by social desirability bias. Also, response rate on the surveys was low, ranging from 25% to 36% of those enrolled, which is an area of improvement for program process evaluation efforts.

## Conclusion

Brighter Bites obtains and distributes donated produce by partnering with local food banks, making it available to families in need, and combines it with experiential evidence-based nutrition education to improve child and parent dietary habits. By implementing the program as a coordinated school health model, leveraging existing expertise of local food banks, and using innovative strategies such as a food co-op concept, Brighter Bites provides consistent opportunities for children to practice healthy behaviors in school and at home.
